# Pre-Practice Hydration Status in Soccer (Football) Players in a Cool Environment

**DOI:** 10.3390/medicina54060102

**Published:** 2018-12-05

**Authors:** Urmo Kiitam, Lilita Voitkevica, Saima Timpmann, Inese Pontaga, Jaan Ereline, Eve Unt, Vahur Ööpik

**Affiliations:** 1 Faculty of Medicine, University of Tartu, 50090 Tartu, Estonia; ukiitam@ut.ee; 2 Department of Anatomy, Physiology, Biochemistry and Hygiene, Latvian Academy of Sport Education, LV-1006 Riga, Latvia; lilita.voitkevica@ikvd.gov.lv (L.V.); inesepontaga@inbox.lv (I.P.); 3 Institute of Sport Sciences and Physiotherapy, University of Tartu, 50090 Tartu, Estonia; saima.timpmann@ut.ee (S.T.); jaan.ereline@ut.ee (J.E.); 4 Department of Cardiology, University of Tartu, 50090 Tartu, Estonia; eve.unt@kliinikum.ee; 5 Department of Sports Medicine, University of Tartu, 50090 Tartu, Estonia; 6 Sports Medicine and Rehabilitation Clinic, Tartu University Hospital, 50406 Tartu, Estonia

**Keywords:** soccer, hypohydration, urine specific gravity, wintertime training

## Abstract

*Background and Objectives:* Only a few studies have reported the pre-practice hydration status in soccer players (SPs) who train in a cool climate. The primary purpose of this study was to examine the hydration status of male semiprofessional SPs immediately before their regular training session in winter. The secondary purpose was to compare the urinary indices of the hydration status of Estonian and Latvian SPs. *Materials and Methods:* Pre-training urine samples were collected from 40 Estonian (age 22.1 ± 3.4 years, soccer training experience 13.7 ± 3.9 years) and 41 Latvian (age 20.8 ± 3.4 years, soccer training experience 13.3 ± 3.0 years) SPs and analyzed for urine specific gravity (U_SG_). The average outdoor temperature during the sample collection period (January–March) was between −5.1 °C and 0.2 °C (Estonia) and −1.9 °C and −5.0 °C (Latvia). *Results:* The average pre-training U_SG_ of Estonian and Latvian SPs did not differ (*P* = 0.464). Pooling the data of Estonian and Latvian SPs yielded a mean U_SG_ value of 1.021 ± 0.007. Hypohydration (defined as a U_SG_ ≥ 1.020) was evident altogether in fifty SPs (61.7%) and one of them had a U_SG_ value greater than 1.030. *Conclusions:* Estonian and Latvian SPs do not differ in respect of U_SG_ and the prevalence of pre-training hypohydration is high in this athletic cohort. These findings suggest that SPs as well as their coaches, athletic trainers, and sports physicians should be better educated to recognize the importance of maintaining euhydration during the daily training routine in wintertime and to apply appropriate measures to avoid hypohydration.

## 1. Introduction

The disadvantageous effects of dehydration during exercise have been well-known for years. Dehydration may impair cardiovascular and thermoregulatory function, increase ratings of perceived exertion, and reduce working capacity [1,2]. The magnitude of these effects has been shown to be correlated to the degree of dehydration [2,3]. Similarly, pre-exercise hypohydration may result in a lower sweat rate, a more rapid rise in core body temperature and a higher heart rate during workout [2,3]. Therefore, the pre-exercise hydration status of athletes has been a topic of several studies [4,5,6,7,8,9,10,11,12,13] and the importance of adequate hydration prior to training has been stressed by the American College of Sports Medicine [14] and National Athletic Trainers’ Association [15].

Urine specific gravity (U_SG_) and urine osmolality (U_OSM_) are used as indices of the hydration status. A strong correlation (*r*-value in the range of 0.916–0.995) has been observed between U_SG_ and U_OSM_ [5,16,17]. The widely accepted U_SG_ cut-off value for the detection of dehydration is ≥1.020 [14,18]. Studies employing this cut-off value have demonstrated that as many as 46% of recreational exercisers [16] and 52–77% of athletes [4,8,9,10,11] could be classified as hypohydrated before a training session or competition. The suggested U_OSM_ cut-off value is more variable, ranging from >586 mOsm/kg to >800 mOsm/kg [6,14,19].

However, marked intercultural and ethnic differences in urinary parameters may occur. The mean U_OSM_, for example, has been reported to be 900 mOsm/kg in Japan and 801 mOsm/kg in Germany, but only 392 mOsm/kg in Poland and in Kenya [20]. Similarly, black women and men exhibited significantly greater U_OSM_ than white women and men living in the same area [21]. Therefore, a certain caution seems to be warranted while using common cut-off values of urine U_OSM_ and U_SG_ for assessing hydration status of individuals living in different cultural environments. 

To date, very little research has been reported on the hydration status of athletes in Estonia [17] and Latvia [22]. This paper reports data on the pre-training hydration status in semiprofessional soccer (football) players (SPs) training in one Estonian and one Latvian city in the winter time. In many countries, including Estonia and Latvia, due to geographical location, SPs have to train in cool environments for several months each year. The study was undertaken considering the fact that the literature regarding pre-practice hydration status in SPs training in a cool climate is scarce and reveals a rather high prevalence of hypohydration among professional [7] and semiprofessional athletes [22]. On the other hand, in cool climatic conditions, soccer training has been shown to induce sweat losses and dehydration similar to that observed in much warmer environments [7]. Furthermore, there is evidence that even mild dehydration may impair a number of physiological functions and deteriorate the soccer-specific physical [23,24] and decision-making [25] performance. Therefore, the main purpose of this study was to examine the hydration status of male semiprofessional SPs immediately before their regular training session in winter. In addition, we aimed to compare the urinary indices of the hydration status of Estonian and Latvian SPs.

## 2. Materials and Methods

### 2.1. Study Design

This was a cross-sectional observational study which was conducted in two locations: Riga (Latvia) and Tartu (Estonia). The study was carried out in two phases. In the preparatory phase, 58 pre-training urine samples collected from Latvian SPs were analyzed for U_SG_ with two different refractometers. The U_OSM_ of the collected urine samples was measured in Tartu using a single unit of freezing point depression osmometer. This preliminary work was undertaken to determine whether the U_SG_ values measured with the two different apparatuses were comparable.

In the main phase of the study, pre-training urine samples were collected from 41 Latvian and 40 Estonian semiprofessional SPs and analyzed for U_SG_ using the aforementioned two refractometers, one in Riga and the other in Tartu.

### 2.2. Participants

In the preparatory phase of the study, 58 male SPs from two teams of the Premium League and one team of the First League of the Latvian Football Federation participated. Their pre-training urine samples were collected within two days at the end of August and the beginning of September 2012. The average ambient temperature in Riga in August and September was 16 °C and 13 °C, respectively.

In the main phase of the study, the Estonian participants (*n* = 40) were male SPs belonging to two teams, one of which was playing in the Premium League and the other in the First Division of the Estonian Football Association. The Latvian participants (*n* = 41) were from one Premium League and from one First League club of the Latvian Football Federation. Urine samples were collected during the period from January to March 2013 in both locations. The average ambient temperature in that period was between −5.1 °C and 0.2 °C (Tartu) and −1.9 °C and −5 °C (Riga).

In both the preparatory and main phases of the study, researchers arrived at the training facilities of the teams before a regular training session, having previously entered into an agreement with the coaches. The players had no prior knowledge of the study in order to avoid affecting their regular pre-training behavior. Upon arrival at the training facility, the players were given a short but complete explanation about the aims and procedures of the study and invited to participate on a voluntary basis. The refusal rate was 16% in Latvia and 11% in Estonia. All subjects who agreed to participate received a written description of the study and were required to provide written informed consent. The protocol of the study was approved by the Research Ethics Committee of the University of Tartu (Estonia), protocol 208/T-6, 17.10. 2011, and by the Research Ethics Committee of the Latvian Academy of Sports Education (Latvia), protocol 2/42722, 29.06.2012.

### 2.3. Procedures

After agreeing to participate in the study, the subjects were measured for height to the nearest 0.5 cm using stadiometer and aligning the head of the subject in Frankfort horizontal plane. The body mass (to the nearest 0.1 kg; wearing briefs) and body fat percentage values were measured using two separate units (one in Tartu and the other in Riga) of the Body Composition Analyzer BC-418 manufactured by Tanita Corporation, Tokyo, Japan. In addition, the subjects completed a short survey on their training experience and typical training regime. After completing the survey, immediately before the beginning of the training session, the subjects donated a urine specimen in a sample cup and delivered it to the researchers. At that point, the subjects had completed the study. The urine samples were placed in a cooling box and transported to the laboratory.

In the preparatory phase of the study in Riga, U_SG_ was measured within three hours after collection using a PAL-10S refractometer (Atago, Minato, Japan). The samples were then frozen and stored at −20 °C. Approximately one month after collection, the frozen samples were packed into a box filled with dry ice and transported from Riga to Tartu. The samples were thawed at room temperature and measured again for U_SG_ using the same unit of PAL-10S refractometer which was previously used in Riga and with a PDX-CL refractometer (VEE GEE Scientific Inc., Kirkland, WA, USA) used only in the Tartu laboratory. In addition, the U_OSM_ was measured using a freezing point depression osmometer (Model 3250; Advanced Instruments Inc., Norwood, MA, USA).

In the main phase of the study, urine samples were frozen after collection and measurement for U_SG_ after thawing at room temperature within one month after collection, using the PAL-10S and PCX-CL refractometers in Riga and Tartu, respectively.

The U_SG_ measuring range of the refractometer PAL-10S is 1.000–1.060, with a resolution of 0.001 and an accuracy of ±0.001. The refractometer PDX-CL possesses a temperature compensation function, a U_SG_ measurement range of 1.0000–1.0500, resolution of 0.0001 and accuracy of ±0.0003. The refractometers were calibrated with distilled water before each use and repeated calibration was performed after every ten samples. The used osmometer is periodically calibrated by a technical service engineer. In addition, the calibration of the device was checked before each use with standard calibration solutions.

U_SG_ values of 1.010–1.020 are considered consistent with euhydration according to the American College of Sports Medicine [14] and National Collegiate Athletic Association [8] criteria, but indicate minimal hypohydration compared to the National Athletic Trainers’ Association’s more detailed classification system [15]. In the current paper, we classify our SPs with U_SG_ ≤ 1.020 as “euhydrated”, those with U_SG_ 1.021–1.030 as “hypohydrated”, and those with U_SG_ > 1.030 as “seriously hypohydrated”.

### 2.4. Statistical Analysis

The SPSS version 20 software was used for performing statistical analysis of the data. The mean values and standard deviations were calculated for all characteristics. The normality of all datasets was examined using the Kolmogorov–Smirnov test. The U_SG_ values measured in fresh and thawed samples by means of two different refractometers were compared using the repeated measures analysis of variance (ANOVA). Student’s independent samples *t*-test was employed to determine differences between Estonian and Latvian SPs regarding their age, anthropometric parameters, training experience, and U_SG_ values. An χ^2^ analysis was performed to compare the prevalence of the hypohydration status among Estonian and Latvian SPs. Linear correlation analysis was used to determine the relationships between various characteristics. A regression analysis model was used to evaluate associations of U_SG_ values measured in the set of fresh samples by means of the PAL-10S refractometer with U_SG_ values measured in the same set of thawed samples with both the PAL-10S and PDX-CL refractometers. Statistical significance was set at *P* < 0.05.

## 3. Results

### 3.1. Preparatory Phase

Data regarding age, anthropometric characteristics, and training experience of the participants in the preparatory phase of the study are presented in Table 1.

The U_SG_ values measured in fresh samples with PAL-10S were significantly greater than those measured in the same set of samples in the thawed state with the same device or with PDX-CL (Table 2). U_OSM_ was only measured in the thawed samples and was 771 ± 259 mOsm/kg. Strong correlations occurred between the U_SG_ values measured in the set of fresh samples by means of the PAL-10S refractometer and the values measured in the same set of thawed samples with both PAL-10S and PDX-CL refractometers (*r* = 0.991; *P* < 0.001 in both cases). Likewise, U_OSM_ only measured in thawed urine samples strongly correlated with the U_SG_ values measured both in fresh (*r* = 0.979; *P* < 0.001) and thawed samples (*r* = 0.974; *P* < 0.001 while using PAL-10S, and *r* = 0.976; *P* < 0.001 while using PDX-CL device).

### 3.2. Main Phase

The descriptive data of the participants in the main phase of the study from both Estonia and Latvia are summarized in Table 3. Estonian and Latvian athletes did not differ in respect of age, anthropometric parameters, or training experience.

As the U_SG_ was only measured in thawed samples in this phase of the study, both Estonian and Latvian data were recalculated into respective fresh urine values on the basis of the results of the regression analysis carried out in the preparatory phase (Table 4).

**Figure 1:**
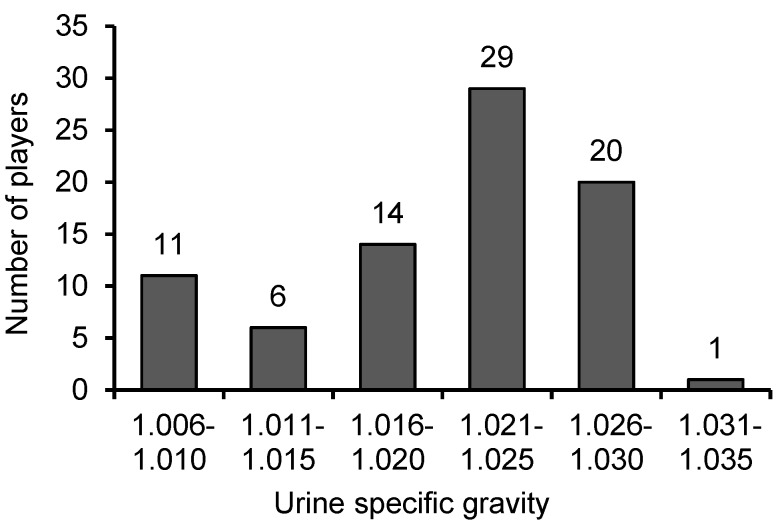
The frequency distribution of the U_SG_ values in the pooled data set of Estonian and Latvian soccer players.

Thus, the average pre-training U_SG_ of Estonian and Latvian SPs was 1.021 ± 0.006 and 1.021 ± 0.007, respectively (*P* = 0.464). The prevalence of hypohydration among Estonian (57.5%) and Latvian (65.9%) SPs was similar (χ^2^ = 0.598; *P* > 0.05). Pooling the data of Estonian and Latvian athletes yielded a mean U_SG_ value of 1.021 ± 0.007. Figure 1 shows the frequency distribution of the U_SG_ values in the pooled data set. Fifty subjects (61.7%) had a U_SG_ value equal to or greater than 1.020 and one subject (1.2%) had a U_SG_ value over 1.030.

## 4. Discussion

The main findings of this study are that pre-training U_SG_ in Latvian and Estonian semiprofessional male SPs did not differ and that the prevalence of pre-training hypohydration evaluated on the basis of U_SG_ was approximately 62% in this athletic cohort in wintertime.

As the main aim of the study was to examine the hydration status of male SPs immediately before their regular training session in winter, we considered it necessary to compare the two ethnically different subgroups of athletes in respect of their U_SG_ before pooling their data. The necessity for such kind of comparison proceeds from the fact that marked intercultural and ethnic differences in urinary parameters may occur [20,21]. For example, in the geographically close countries of Germany and Poland, in males and females of similar age groups, mean U_OSM_ values of 801 mOsm/kg and 392 mOsm/kg have been reported, respectively [20]. The existence of large intercultural differences in urine parameters related to hydration status may be based on unique regional customs regarding preferred beverages and food items [20,26]. Thus, the identical mean U_SG_ values determined in Latvian and Estonian participants in this study suggest that generally their nutritional preferences and behavior were similar and, therefore, pooling their urine data for assessing hydration status is acceptable.

There are very few studies to date that have assessed the pre-practice hydration status in SPs who train in a cool climate. Employing the U_SG_ cut-off value of ≥1.020 Ozolina et al. [22] reported a high prevalence of pre-training hypohydration (65%) in male SPs possessing similar to the participants in the current study athletic qualification, and Maughan et al. [7] observed mild hypohydration (defined as U_OSM_ above 900 mOsm/kg) in 35% of the elite male SPs. Gibson et al. [27] found that 45% of female Canadian junior elite soccer athletes presented to practice in a hypohydrated state (U_SG_ > 1.020). In light of these limited data, the notably high (approximately 62%) prevalence of pre-training hypohydration in the participants in the current study is not surprising. Interestingly, Silva et al. [9] monitored elite male Brazilian adolescent SPs during three consecutive training days in the heat (27.6 °C–33.1 °C) and observed the occurrence of pre-practice hypohydration in 45–85% of athletes in different days. Similarly, Arnaoutis et al. [28] found that 90% of adolescent SPs began their training session in the hypohydrated state in the heat (27.2 °C). In temperate climates (10 °C–23 °C), an even higher (98%) prevalence of moderate to severe hypohydration before regular training practice was observed in professional Chilean SPs [29]. However, in similar climatic conditions (21.3 °C), Ersoy et al. [30] only reported the risk of low-level hypohydration in young elite SPs during 3-day preparation for an important competitive match. Thus, most of the relevant literature [7,9,22,27,28,29] and the data of the current study suggest that in SPs of different qualification maintaining euhydration during daily training routine may be equally challenging in cool, temperate, and warm climates.

According to Shirreffs [31], in order to avoid a decrease in physical performance, SPs should limit the degree of dehydration to less than 2% of body mass loss, whereas a recent excellent review [32] concluded that hypohydration typically impair cognition, technical skills, and physical performance at higher levels of body mass loss (3–4%) in team sports athletes, including SPs. Maughan et al. [7] reported that SPs who started a training session in the cold with a relatively high U_OSM_ tended to drink more water during the session than others, but this did not prevent the significant body mass loss revealing dehydration of approximately 1.6% over the training session. Not surprisingly, Duffield et al. [33] observed a much greater degree of dehydration (2.1–3.5%) in professional SPs over a training session in the heat, and Arnaoutis et al. [28] reported that drinking ad libitum during practice did not prevent the further dehydration of adolescent players who began training in the hypohydrated state. These findings suggest that SPs are not able to compensate sweat losses with fluid consumption during training neither in warm nor in cool environments. Therefore, in players who are hypohydrated before training, the cumulative water deficit developing during a training session and concomitant decrease in performance could be expected to be greater than in players who start training in a euhydrated status. In addition, recent findings suggest that even mild pre-match dehydration increases the physiological stress during a game as revealed by significant increases in cortisol levels in young elite SPs [34] and that a small degree of hypohydration induced by moderate exercise and fluid restriction impair the endothelial function [35]. In the long-time perspective, pre-practice hypohydration could lead to a suboptimal training load tolerance and reduce the efficacy of the whole training process. It is relevant to note that experienced physiotherapists working in Brazilian soccer clubs consider hydration status as one of the “very important” or “important” injury risk factors [36]. Furthermore, frequent episodes of the occurrence of persistent water deficit in the body during daily training routine could cause a situation similar to chronic hypohydration. Chronic hypohydration and inadequate fluid consumption have been linked to chronic illnesses [37].

Thus, it is evident that the maintenance of euhydration of professional and semiprofessional SPs, including those who train in cool environments, deserves the serious attention of the athletes themselves, but also of the coaches, athletic trainers, and sports physicians. Maintaining body fluid balance in cool climate could be an equally great challenge as in temperate or warm environments because of the changed thirst sensation. Kenefick et al. [38] reported that when either euhydrated or hypohydrated, thirst at rest and during moderate-intensity exercise was attenuated by up to 40% in cool compared to temperate environments in young men. The findings of Maughan et al. [7] who compared sweat losses and fluid intake in football players training in temperate (25 °C, relative humidity 60%) and cool (5 °C, relative humidity 81%) environments, corroborate the involvement of attenuated thirst sensation in developing acute training-induced dehydration in cold. Specifically, they found that sweat losses were rather similar in the two environments (difference less than 9%), but fluid intake in cool conditions was merely about 50% of that in temperate environments [7]. Thus, the high prevalence of pre-practice hypohydration observed in the current study and reported in pervious papers [7,22] could partly be explained by attenuated thirst sensation in cold environments. This may favor the development of acute dehydration during a training session and hinder rehydration during recovery between consecutive training sessions.

Nichols et al. [39] studied collegiate athletes and reported significant positive correlations between knowledge, attitudes, and behaviors regarding hydration and fluid replacement in sports training. O’Reilly and Wong [40] instructed the SPs participating in their study to drink at least 500 mL of water in the two hours prior to each training session to increase the likelihood of commencing training in a euhydrated state. Indeed, they found that each participant reported to the laboratory in a euhydrated condition (U_SG_ ˂ 1.020). Collectively these findings [39,40] reveal the importance of relevant knowledge and suggest that rather simple but appropriate counseling may turn out to be effective in improving the athletes’ hydration status.

An important methodological aspect of this study is that urine samples collected in the main phase were initially frozen and measured for U_SG_ after thawing at room temperature. Others [41] have evaluated the hydration status in athletes on the basis of U_OSM_ measured in thawed urine specimens. However, Adams et al. [42] have shown that a single freezing/thawing cycle lowers both U_SG_ and U_OSM_ values. We also observed a small but statistically significant decline while comparing the U_SG_ values of fresh and thawed samples collected in the preparatory phase of the study. A small but statistically significant difference was also evident between U_SG_ measures taken with two different refractometers. Therefore, considering these findings, in the main phase of the study, both Estonian and Latvian U_SG_ data were recalculated into respective fresh urine values on the basis of the results of regression analysis carried out using the preparatory phase data. According to Armstrong et al. [43], U_SG_ and U_OSM_ strongly correlate (*R*^2^ = 0.81–0.91) and may be used interchangeably to assess hydration status. While these researchers [43] analyzed fresh urine, we observed similar strong relationships between U_OSM_ measured in thawed samples and U_SG_ measured with PAL-10S in fresh and thawed samples (*R*^2^ = 0.96 and 0.95, respectively) and with PDX-CL in thawed samples (*R*^2^ = 0.95). Thus, based on the considerations presented in this paragraph, we are confident that we correctly recalculated the U_SG_ of thawed samples into U_SG_ values of fresh samples.

An acknowledged limitation of this study is using the spot sample collection protocol as these samples may not represent the true 24-h void [44]. However, collecting an upon-waking mid-flow urine sample from a fasted and rested athlete that is considered the preferred protocol [44] was not possible by logistical reasons. It is also relevant to note that generalizability of our findings to the rest of Latvian and Estonian semiprofessional SPs requires some caution because a limited number of athletes was studied.

## 5. Conclusions

In conclusion, the results of the study reveal that Estonian and Latvian SPs do not differ in respect to U_SG_ and that the prevalence of pre-training hypohydration is high in this athletic cohort. These findings suggest that SPs as well as their coaches, athletic trainers, and sports physicians should be better educated to recognize the importance of maintaining euhydration during the daily training routine in wintertime and to apply appropriate measures to avoid hypohydration.

## Figures and Tables

**Table 1:** The characteristics of the participants in the preparatory phase of the study (*n* = 58).

Parameters	Mean ± SD
Age (years)	22.0 ± 2.8
Height (m)	1.82 ± 0.06
Body mass (kg)	76.7 ± 7.9
Body mass index (kg/m^2^)	23.2 ± 1.7
Body fat (%)	10.9 ± 3.7
Training volume (h/week)	9.6 ± 0.6
Training experience (years)	13.0 ± 3.1

**Table 2:** The U_SG_ values measured in the same set of urine samples (*n* = 58) in fresh and thawed conditions using two different refractometers (PAL-10S and PDX-CL).

PAL-10S(fr)	PAL-10S(th)	PDX-CL(th)
1.020 ± 0.007	1.019 ± 0.008 *#	1.018 ± 0.007 *

Values are expressed as mean ± SD. PAL-10S and PDX-CL, refractometers; (fr), fresh sample; (th), thawed sample. Significantly different (*P* < 0.05): * from PAL-10S(fr); # from PDX-CL(th).

**Table 3:** The characteristics of the participants in the main phase of the study.

Parameters	EST, *n* = 40	LAT, *n* = 41	*P*
Age (years)	22.1 ± 3.4	20.8 ± 3.4	0.091
Height (m)	1.81 ± 0.06	1.82 ± 0.07	0.511
Body mass (kg)	76.8 ± 10.7	76.8 ± 8.0	0.998
Body mass index (kg/m^2^)	23.4 ± 2.1	23.2 ± 1.7	0.118
Body fat (%)	11.3 ± 3.7	10.5 ± 3.3	0.298
Training volume (h/week)	9.9 ± 3.2	9.3 ± 0.8	0.272
Training experience (years)	13.7 ± 3.9	13.3 ± 3.4	0.617

Values are expressed as mean ± SD. EST, Estonian soccer players; LAT, Latvian soccer players.

**Table 4:** The results of the regression analysis of U_SG_ values measured in fresh samples (*n* = 58) by means of the PAL-10S refractometer and in the same set of thawed samples by means of both PAL-10S and PDX-CL refractometers.

	*R* ^2^	*F*	SEE	*P*
PAL-10S(th)	0.982	3081	0.0009	<0.001
PDX-CL(th)	0.983	3190	0.0009	<0.001

(th), thawed sample.
